# Menaquinone biosynthesis in *Lactococcus lactis*: Recent advances and future prospects

**DOI:** 10.1016/j.engmic.2026.100304

**Published:** 2026-09-02

**Authors:** Yu Lou, Jingjing Shuang, Jian Kong, Tingting Guo

**Affiliations:** State Key Laboratory of Microbial Technology, Shandong University, No. 72 Binhai Road, Qingdao, 266237, China

**Keywords:** Menaquinone, Vitamin K2, *Lactococcus lactis*, Biosynthesis

## Abstract

Menaquinone, also known as vitamin K2, has extensive nutritional and pharmaceutical applications, such as in bone health maintenance and cardiovascular protection. It consists of a naphthoquinone ring and a side chain with varying numbers of isoprenoid units; hence, it is abbreviated as MK-n, where n represents the number of isoprenoid units. Since long-chain MKs (n = 5–13), which are produced only by bacteria, exhibit longer half-lives and higher bioavailability than short-chain MKs, the microbial production of long-chain MKs has attracted great interest in the fields of food microbiology and synthetic biology. *Lactococcus lactis*, a key starter strain for dairy processing, is an excellent candidate as it produces MK-8, MK-9, and MK-10. In recent years, research on MK biosynthesis in *L. lactis* has gained increasing attention, and improvements in MK production have been achieved through physiological exploration, fermentation optimization, and metabolic engineering. This review summarizes the MK biosynthetic pathway, its physiological functions, and regulation of the MK profile as well as the production titer in *L. lactis*. Moreover, it discusses the high-yield schemes of MK in *Escherichia coli* and *Bacillus subtilis* to provide insights for developing *L. lactis* as an MK-producing cell factory.

## Highlights

• The microbial production of menaquinones (MKs) have attracted increased attention.
• *Lactococcus lactis* is an excellent bacterial chassis for MK production in bacteria.
• Methods to increase MK biosynthesis are being developed in L. *lactis*.
• Recombineering is a promising tool to increase MK titers in L. *lactis*.
• Insights from MK production in *Escherichia coli* and *B. subtilis* are used for L. *lactis*.

## Graphical abstract

**Figure:**
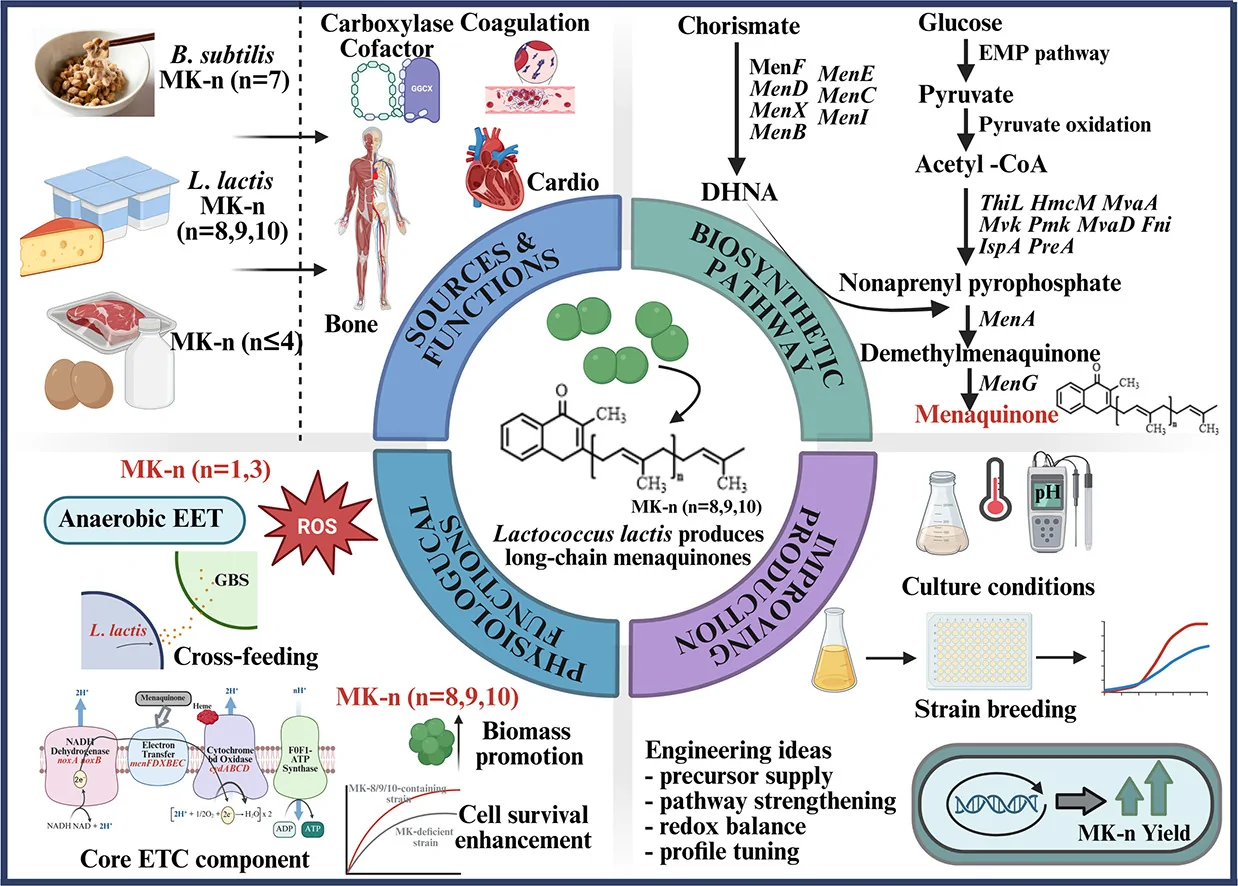

## 1 Introduction

**Fig. 1:**
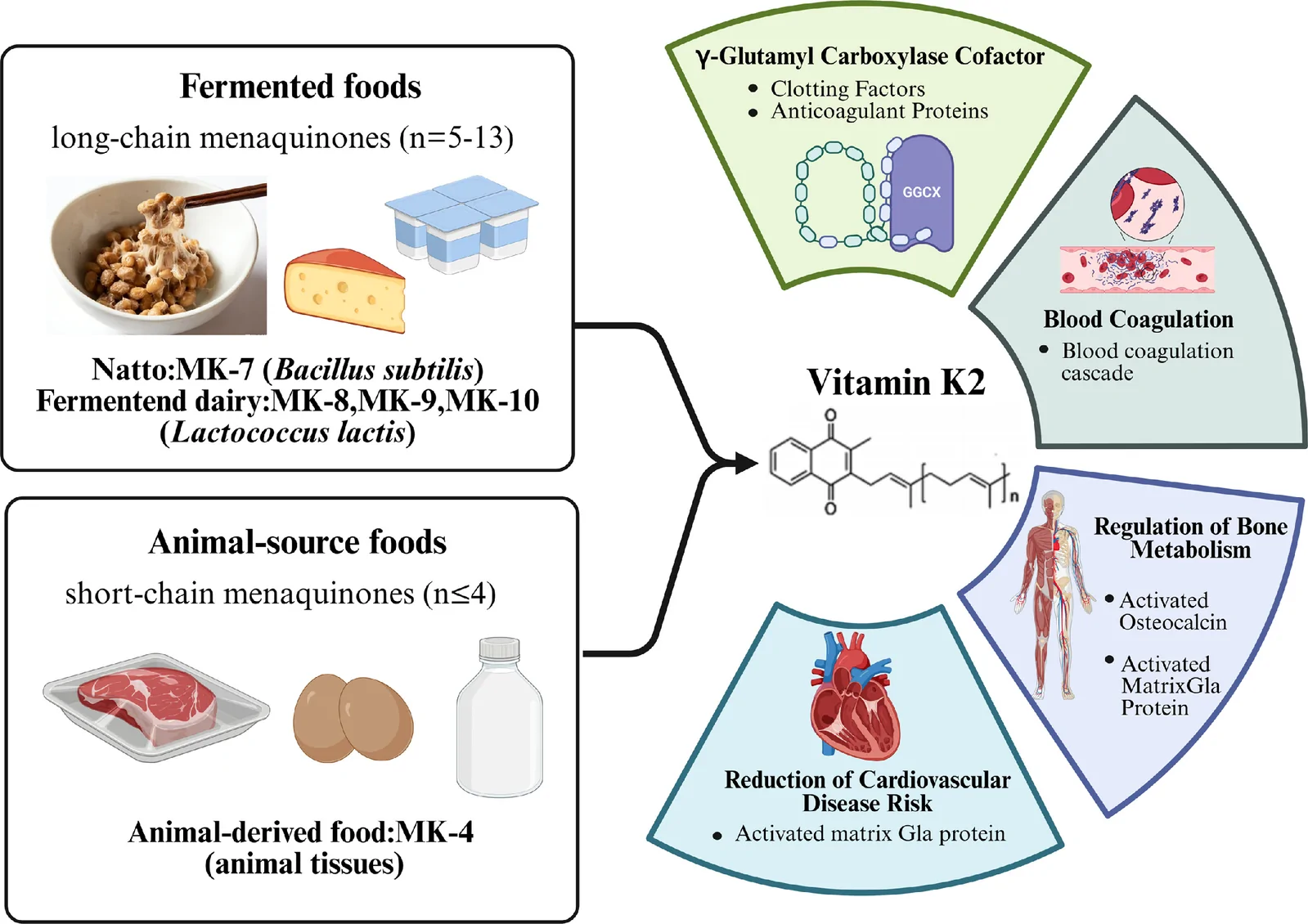
Sources and biological functions of Vitamin K2.

Vitamin K (VK) is an essential fat-soluble vitamin in humans; it includes a group of closely related derivatives with a 2-methyl-1,4-naphthoquinone ring as its basic head structure. VK is mainly classified into two categories: VK1 (phylloquinones) and VK2 (menaquinones, MKs) [[1], [2], [3]]. Characterized by a phytyl side chain, VK1 is ubiquitous in green leafy vegetables and vegetable oils and is the primary dietary source of VK [2,4]. In contrast, VK2 contains a polyisoprenoid side chain, and is further divided into short-chain MKs (n ≤ 4) and long-chain MKs (n = 5–13), depending on the number of isoprenoid residues (n) in its side chain (Fig. 1). Short-chain MKs can be endogenously synthesized in human and animal tissues and are abundant in meat, eggs, and dairy products. In contrast, long-chain MKs, which are exclusively produced by bacteria, are considered to have longer half-lives and better bioavailability owing to their more hydrophobic nature than short-chain MKs [3].

As a critical cofactor for γ-glutamyl carboxylase, VK2 plays a pivotal role in blood coagulation and the regulation of bone metabolism, and can also effectively mitigate vascular calcification [1]. Osteocalcin is carboxylated by γ-glutamyl carboxylase, which enables it to bind calcium ions. Without sufficient VK2, osteocalcin cannot be activated and calcium cannot be effectively deposited into bones, increasing the risk of osteoporosis [5,6]. In contrast, clinical studies have confirmed a negative correlation between dietary VK2 intake and coronary artery calcification, where individuals in the high intake group presented with a lower degree of calcification. A daily VK2 intake of at least 32.7 µg was associated with a 41% reduction in the incidence of coronary heart disease (CHD); an additional 10 µg of daily VK2 intake contributed to a further 9% decrease in CHD risk [7]. VK2 can be supplemented by fermented foods such as natto and cheese [6,8]. Japan has pioneered the development and application of VK2, a breakthrough closely tied to natto, which is rich in VK2, containing approximately 0.7–1.0 mg of VK2/100 g natto [9,10]. However, it is not easy to consume natto because of its flavor. At present, inadequate dietary VK2 intake is a global issue. Even in Western European countries, where dairy product consumption is relatively high, dietary VK2 intake only accounts for 10% to 25% of the total vitamin K needed [11,12]. Therefore, developing food starters with enhanced VK2 biosynthesis capacity holds great practical significance.

MK is an essential component of bacterial cell membranes and plays crucial roles in oxidative phosphorylation, electron transport in the respiratory system, and active cellular transport [13]. A wide variety of bacteria can produce MK, which is primarily distributed among *Bacillus, Staphylococcus, Bacteroides, Lactococcus*, and *Leuconostoc* species [14]. The biosynthetic pathway of long-chain MK has been thoroughly studied using *B. subtilis* as a model; the metabolic engineering of *B. subtilis* enables the production of MK-7 at titers of up to 415 mg/L [15]. Currently, *B. subtilis* is widely employed as a microbial cell factory for vitamin K2 (MK-7) production because of its generally recognized as safe (GRAS) status, food safety, well-established genetic engineering tools, and mature fermentation and downstream extraction processes [[16], [17], [18]]. However, as a fermented food carrier of *B. subtilis* rich in VK2, natto has a relatively limited audience. Another microbial cell factory for VK2 is *E. coli*, which has a fully elucidated genetic background and a well-established cultivation regulation system; however, *E. coli* poses the risk of residual lipopolysaccharide endotoxins, prohibiting its deployment in food-related manufacturing [[19], [20], [21]]. The major dairy starter, *L. lactis* is a well-known natural producer of long-chain MKs, particularly MK-8, MK-9, and MK-10. However, the long-chain MK yield of *L. lactis* is lower than that of *B. subtilis*, where the median contents of naturally occurring MK-9 and MK-8 in semi-hard cheeses are about 149 µg/100 g and 55 µg/100 g, respectively [22]. A comparison of the three possible chassis types for the VK is presented in Table 1. With in-depth research on the physiological functions of *L. lactis* and the improvement of its genetic manipulation tools, the development of *L. lactis* strains with higher VK2 production has attracted increasing attention [[23], [24], [25]], bringing about the prospect of *L. lactis* as a VK2 chassis. In this review, we first elaborate on the core metabolic traits of *L. lactis*, followed by a systematic dissection of the biosynthetic pathways and physiological roles of MK in *L. lactis* as well as the selection of *L. lactis* strains with improved production of long-chain MKs. The high-yield strategies of VK2 developed in other microbial chassis (e.g., *E. coli* and *B. subtilis*) were also integrated, guiding a theoretical framework and providing technical insights for the rational metabolic engineering of *L. lactis* to further improve VK2 production.

**Table 1:** Comparison of the characteristics of three MK chassis.

Chassis Strain	Applicable Scenarios	Advantages	Disadvantages
*Escherichia coli*	Biosynthesis of high-value MK products and construction of engineered microbial cell factories [20,26,27]	Well-elucidated genetic background; mature genetic engineering toolkit; well-defined metabolic network; fast growth and mature fermentation technology [19,20,28,26,27,29]	Endotoxin risk; limited food-grade application; high-cost anaerobic MK synthesis; aerobic competing pathways of MK-7; complex MK-7 pathway requiring IPP/DHNA balance [21,27]
*Bacillus subtilis*	Industrial production of food-grade MK [[16], [17], [18]]	GRAS status and absence of endotoxin contamination; well-established MK-7 metabolic engineering strategies; efficient fermentation optimization approaches [30]	Complex metabolic regulation; requirement for multi-module pathway engineering; low native productivity [31,32]
*Lactococcus lactis*	Value-added food products [23,33,34]	GRAS food-safe chassis; facultative anaerobic fermentation with few byproducts; high MK-8/−9/−10 proportion [22,35,36,33]	Relatively limited MK productivity compared with highly engineered industrial hosts; constrained biosynthetic flux by precursor supply and regulation of the MK pathway; further optimization needed for industrial-scale production [14,25]

## 2 Carbon metabolic characteristics of *L. lactis*

*L. lactis* is the most widely used starter strain for cheese production. Comparative genomic analysis indicated that this commercial starter originated from a non-dairy niche and was domesticated in the milk environment through reductive evolutionary processes [37]. Driven by reductive evolution, *L. lactis* has undergone targeted metabolic adaptations to quickly ferment lactose and break down casein [[37], [38], [39]]. *L. lactis* of dairy origin utilizes fewer types of carbon sources compared to plant-derived strains, primarily lactose and glucose, as well as galactose and fructose [39,40]. Lactose is efficiently transported into *L. lactis* cells via the phosphotransferase system and is rapidly hydrolyzed into glucose and galactose-6-phosphate. Glucose, the preferred and fastest-utilized carbon source, directly enters the Emden-Meyerhof-Parnas (EMP) pathway, whereas galactose-6-phosphate is funneled into glycolysis via the Leloir or tagatose-6-phosphate pathway [[39], [40], [41]]. In the presence of multiple carbon sources, *L. lactis* typically prioritizes glucose utilization and initiates the metabolism of alternative substrates only after glucose depletion, which is regulated by a carbon catabolite repression mechanism [40,41].

### 2.1 Homolactic fermentation

**Fig. 2:**
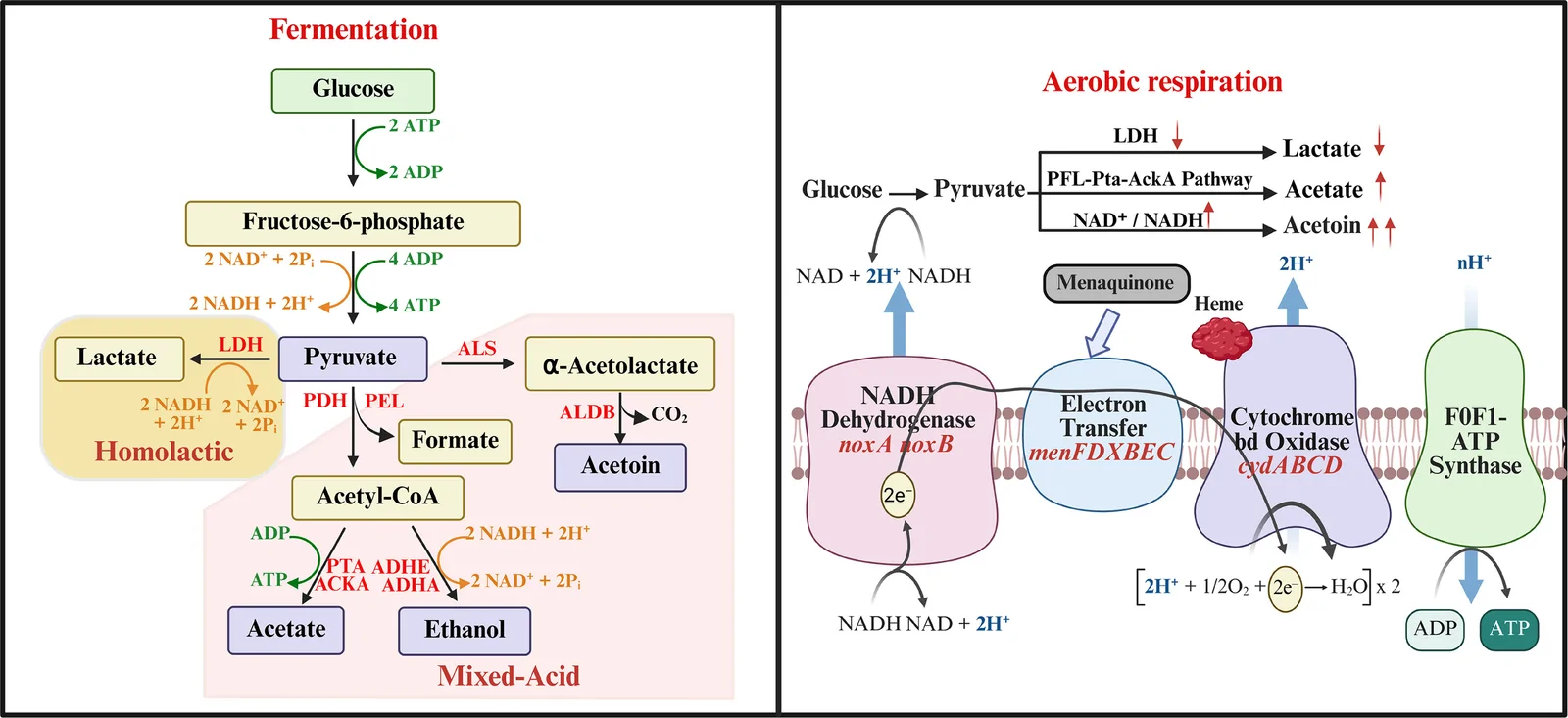
**Metabolic mode of fermentation and aerobic respiration in *L. lactis.*** LDH, lactate dehydrogenase; ALS, α-acetolactate synthase; ALDB, acetolactate decarboxylase; PDH, pyruvate dehydrogenase; PFL, pyruvate formate lyase; PTA, phosphotransacetylase; ACK, acetate kinase; ADHE/ADHA, acetaldehyde dehydrogenase/alcohol dehydrogenase.

Under static conditions, glycolytic carbon flow is highly directed in *L. lactis*, with over 90% of the pyruvate flux ending as lactate, resulting in homolactic fermentation (Fig. 2). Cells obtain only limited energy via substrate-level phosphorylation, and the intracellular redox balance is maintained by lactate dehydrogenase, which consumes the NADH produced during glycolysis.

### 2.2 Mixed-acid fermentation

Under glucose-limited chemostat cultures or low growth rate conditions, pyruvate formate lyase (PFL) is activated, leading to the production of formate, ethanol, and acetate, and bacterial cells undergo mixed-acid fermentation [37,39]. Under aerobic conditions, PFL is inactivated, whereas the pyruvate dehydrogenase complex is activated. Pyruvate is converted into acetyl-CoA by the pyruvate dehydrogenase complex (Fig. 2). Thus, lactate production decreases, whereas acetate production increases significantly, and the strain still undergoes mixed-acid fermentation. Excess pyruvate can also be converted into diacetyl and acetoin [39,42]. In the process of acetate production, acetyl-CoA is first converted into acetyl-Pi by phosphotransacetylase under aerobic conditions, and then acetyl-Pi is further converted into acetate, catalyzed by acetate kinase, along with the production of ATP.

### 2.3 Aerobic respiration

Upon exogenous supplementation with heme (Fig. 2), L*. lactis* initiates aerobic respiration via mixed acid fermentation [43,44]. This metabolic switch relies on three core components: NADH dehydrogenase (encoded by *noxA* and *noxB*), MK (encoded by the *menA* gene and the *menFDXBEC* gene cluster), and cytochrome bd oxidase (encoded by the *cydABCD* gene cluster). Briefly, NADH generated from glycolysis acts as the electron donor and is oxidized to NAD⁺ by NADH dehydrogenase. Electrons are then transferred via MK to cytochrome bd oxidase and finally to oxygen to form water, accompanied by efficient ATP production via the F₀F₁-ATP synthase; this process is far more productive than fermentative metabolism [[45], [46], [47], [48]]. During this process, NADH dehydrogenase competes with lactate dehydrogenase for NADH, resulting in a marked decrease in lactate production, increased acetate formation, and slower environmental acidification. As a result, carbon sources are more efficiently utilized, and biomass is greatly increased [49,50]. Unlike some *Lactobacillus* species that require exogenous MK [51], *L. lactis* possesses a complete MK biosynthetic pathway. As a key intermediate carrier in the electron transport chain, MK enables efficient electron transfer. Its biosynthetic capacity is a prerequisite for aerobic respiration in this organism and is a major metabolic advantage over other lactic acid bacteria.

## 3 MK biosynthesis in *L. lactis*

### 3.1 MK synthetic pathway

As an essential electron transport carrier in the respiratory chain, MK is synthesized by three metabolic modules in *L. lactis*, including 1,4-dihydroxynaphthoic acid (DHNA) synthesis, isoprenoid side chain synthesis, and their polymerization and methylation [23,[28], [35], [52], [53]].

**Fig. 3:**
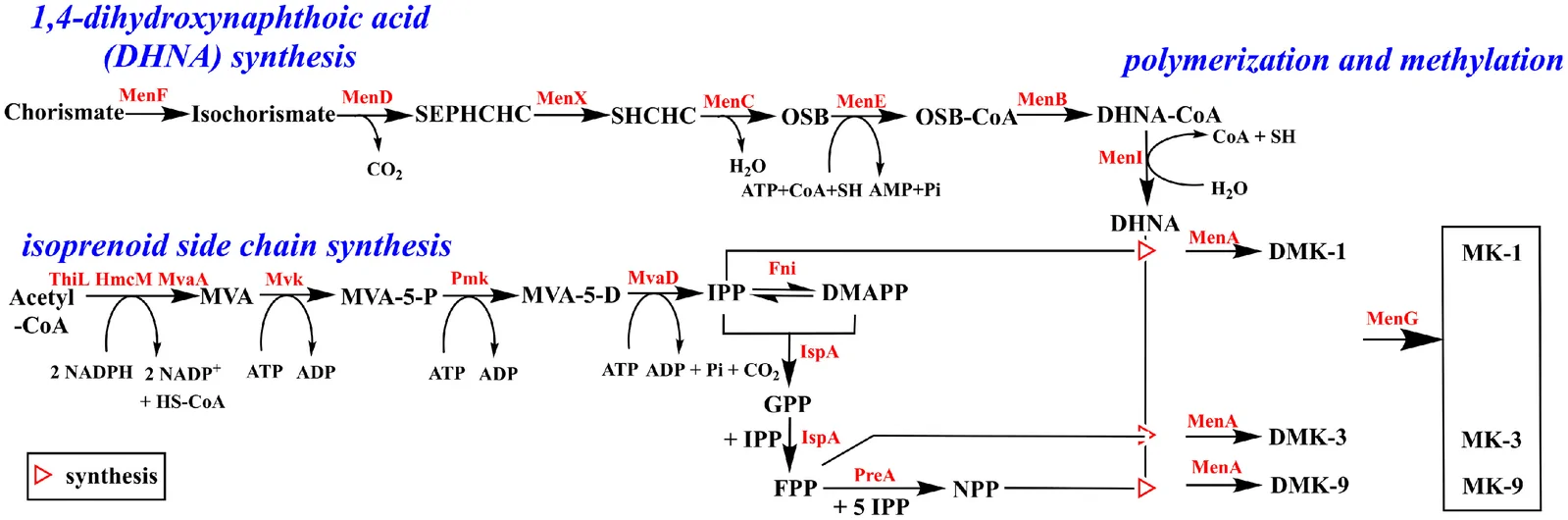
**MK Biosynthetic Pathway in *L. lactis.*Enzymes:** MenF, isochorismate synthase; MenD, 2-succinyl-5-enolpyruvyl-6-hydroxy-3-cyclohexene-1-carboxylate synthase; MenX, menaquinone biosynthesis related protein; MenC, o-succinylbenzoate synthase; MenE, o-succinylbenzoate-CoA ligase; MenB, 1,4-dihydroxy-2-naphthoyl-CoA synthase; MenI, 1,4-dihydroxy-2-naphthoyl-CoA hydrolase; MenA, 1,4-dihydroxy-2-naphthoate heptaprenyltransferase; MenG, demethylmenaquinone methyltransferase; ThiL, thiamine monophosphate kinase; HmcM, 3-hydroxy-3-methylglutaryl-CoA synthase; MvaA, 3-hydroxy-3-methylglutaryl-CoA reductase; Mvk, mevalonate kinase; Pmk, phosphomevalonate kinase; MvaD, diphosphomevalonate decarboxylase; Fni, isopentenyl-diphosphate delta-isomerase; IspA, farnesyl diphosphate synthase; PreA, prenyldiphosphate synthase. **Substrates:** SEPHCHC, 2-succinyl-5-enolpyruvyl-6-hydroxy-3-cyclohexene-1-carboxylate; SHCHC, 2-succinyl-6-hydroxy-2,4-cyclohexadiene-1-carboxylate; OSB, o-succinylbenzoate; OSB-CoA, o-succinylbenzoyl-CoA; DHNA-CoA, 1,4-dihydroxy-2-naphthoyl-CoA; DHNA, 1,4-dihydroxynaphthoic acid; DMK, demethylmenaquinone; MK, menaquinone; MVA, mevalonate; MVA-5-P, mevalonate-5-phosphate; MVA-5-D, mevalonate-5-diphosphate; IPP, isopentenyl diphosphate; DMAPP, dimethylallyl pyrophosphate; GPP, geranyl diphosphate; FPP, farnesyl diphosphate; NPP, nonaprenyl pyrophosphate.

For DHNA formation, the intermediate metabolites phosphoenolpyruvate and erythrose-4-phosphate are used to synthesize shikimic acid, which is further converted to chorismate as the starting substrate of the quinone skeleton of DHNA by a seven-enzyme pathway consisting of MenF, MenD, MenX, MenC, MenE, MenB, and MenI (Fig. 3). First, chorismate is converted into isochorismate by the isochorismate synthase MenF, which is then converted into 2-succinyl-5-enolpyruvyl-6‑hydroxy-3-cyclohexene-1-carboxylate (SEPHCHC), a key intermediate for DHNA, under the catalysis of the SEPHCHC synthase MenD [28,54]. The reaction catalyzed by MenE consumes ATP and completes the construction of the core ring structure [54,55]. A recent report found that MenF and MenD are responsible for the regulation of DHNA biosynthesis, and that MenD plays a more prominent role in controlling DHNA concentrations [23,25,[28], [35], [52], [53]].

Isoprenoid side chain synthesis begins with acetyl-CoA via the mevalonate (MVA) pathway. Acetyl-CoA is condensed and reduced to MVA by the consumption of NADPH. MVA is then converted to isopentenyl diphosphate (IPP, C5) and dimethylallyl diphosphate (DMAPP, C5). Two molecules of IPP and one molecule of DMAPP are then condensed into farnesyl diphosphate (FPP, C15), which is catalyzed by geranylgeranyl diphosphate synthase (IspA). By further adding IPP units to FPP, polyprenyl diphosphate chains of different lengths are formed. For example, one molecule of FPP condenses with four IPP molecules to generate heptaprenyl diphosphate (HPP, C35); this process is catalyzed by heptaprenyl diphosphate synthase, which is encoded by *hepT*/*hepS* in *B. subtilis*. Bøe (2020) reported that nonaprenyl pyrophosphate (NPP, C45) could be produced using five IPP molecules and one FPP molecule by a potential prenyl diphosphate synthase in *L. lactis* [44]; however further biochemical evidence still needs to verify its function.

A polyprenyl diphosphate chain is attached to the C3 position of DHNA by the DHNA polyprenyltransferase MenA to form demethylmenaquinone (DMK), which is further methylated by the methyltransferase MenG. The length of the polyprenyl diphosphate chain determines the type of MK produced. When DHNA is combined with IPP or FPP, MK-1 or MK-3 are produced, respectively; when combined with HPP or NPP, MK-7 or MK-9 are produced [54,[56], [57], [58]].

### 3.2 Physiological functions of MK in *L. lactis*

As an electron carrier in Gram-positive bacteria, MK participates in both aerobic and anaerobic respiration and serves as a key molecule for maintaining bacterial energy metabolism, redox balance, and survival adaptability [47]. MK drives extracellular electron transfer (EET) to support anaerobic respiration and generates reactive oxygen species (ROS) via reduction reactions [59,60]. Simultaneously, MK acts as a critical factor in the aerobic respiratory chain, enhancing ATP synthesis, and optimizing metabolite production [51,[61], [62], [63]]. Given its indispensable role in bacterial respiration, the MK biosynthetic pathway is a novel target for the development of antibacterial drugs [47]. In *L. lactis*, the functions of MK are highly chain length-dependent and environment-specific, exhibiting distinct functional differentiation under aerobic and anaerobic conditions.

MK-1 consists of a DHNA unit and an IPP unit. A *L. lactis* mutant strain with only MK-1 production exhibits higher reduction efficiency toward azo dyes and Cu²⁺ under anaerobic conditions, presenting superior performance of MK-1 in EET. MK-3, which has three IPP units in its side chain, exhibits a function similar to that of MK-1. It has been shown that MK-3 is mainly synthesized in the late growth stage, and its production is directly controlled by the carbon catabolite protein CcpA. Compared with long-chain menaquinones, short-chain menaquinones such as MK-3 exhibit greater efficiency in EET under anaerobic conditions, including Cu^2+^ reduction [35,36]. By supporting anaerobic respiration with MK-1 or MK-3 as electron carriers, cell viability can be enhanced in the stationary phase because additional energy is generated [35,64,65]. Under aerobic culture conditions lacking heme, where the respiratory chain cannot be activated, MK synthesized by *L. lactis* mediates oxygen reduction and generates ROS. Among these quinones, endogenous menaquinones, particularly short-chain forms, such as MK-3 and demethyl-MK-3, can undergo redox cycling with oxygen, promoting ROS formation. Oxidative stress is further exacerbated by redox-active metals, including copper and iron, which catalyze the generation of highly reactive hydroxyl radicals. Consequently, wild-type strains producing short-chain MKs exhibit impaired growth under metal-induced oxidative stress, whereas *menB*-deficient mutants lacking endogenous MK synthesis exhibit enhanced tolerance [35,36]. Moreover, the short-chain MKs produced by *L. lactis* can cross-feed Group B Streptococcus (*Streptococcus agalactiae*), an opportunistic pathogen, to activate respiration and stimulate its growth [36]. Under aerobic respiration conditions, long-chain MKs (MK-8/9) and MK-3 are responsible for electron transport in *L. lactis*; MK-8/9 are the core members of the electron transport chain, giving rise to an increased biomass yield, higher oxygen consumption rate, and longer cell survival [66]. It has been suggested that the difference in hydrophobicity between short- and long-chain MKs is a major determinant of various electron transfer pathways. More hydrophobic long-chain MKs are embedded in the lipid membrane and are likely to participate in aerobic respiration. In contrast, less hydrophobic short-chain MKs are more functional in EET or even act as soluble electron shuttles [60,67].

### 3.3 Regulation of MK profile and production titer in *L. lactis*

As described above, MK homologs with different chain lengths differ in their bioavailability and physiological functions. A large diversity of MK types and levels have been reported in different *L. lactis* strains [45,33]. Short-chain MKs such as MK-1 and MK-3, and long-chain MK-5 to MK-10 can be produced by *L. lactis*, with MK-8, MK-9, and MK-10 being the most abundant forms [45,33]. According to current reports, both the MK profile and total yield can be regulated simultaneously via optimization of culture conditions or strain development (Table 2).

**Table 2:** Overview of menaquinone (MK) production performance and engineering strategies in different microbial strains.

Strain/Host	MK Profile	MK Titer	Main strategy
*Lactococcus lactis*	MK-5 to MK-10	Max titer increased by 5.2-fold compared with the original strain; specific values not reported	Optimize culture conditions for long-chain MK production, including temperature, carbon sources and metabolic modes [68]
	Dominated by MK-1, -3, -8, and -9	Anaerobic: 60 nmol/g dry cell weight (DCW), aerobic: 90 nmol/g DCW, respiratory condition: 70 nmol/g DCW	Elucidate distinct roles of short-chain and long-chain MKs in cellular physiology, membrane fluidity and the respiratory electron transport chain; MKs with different chain lengths differentially regulate oxygen tolerance, extracellular reducing capacity and central carbon metabolism [35]
	MK-3 or MK-9	Total MK level reached nearly 700 nmol/L (500 ng/g fermented milk), with MK-7 to −9 accounting for over 93% of total MK	Overexpress *menF/menA* to redirect metabolism toward short-chain MK-3; overexpress *mvk / llmg_0196* to enhance supply of long isoprenoid side chains and improve long-chain MK-9 production [23]
	MK-9 (65–70%), MK-8 (20%), MK-3 (5–9%)	Titer increased by 50–110% compared with the original strain (80 ± 19 nmol/g DCW)	Adaptive laboratory evolution via 100 consecutive passages under aerobic conditions (mutations in *ldh* and *gapB*) [24]
	MK-8, MK-9	Total MK: 320 µg/kg cheese; MK-9, 149 µg/kg cheese; MK-8, 70 µg/kg cheese	Evaluate native MK-producing capacity and distribution profiles of diverse *Lactococcus* strains during cheese fermentation [22]
	MK-8, MK-9	MK-8, 71 nmol/L, MK-9, 113 nmol/L	Naturally high-yield wild strains isolated from fermented milk [33]
*Escherichia coli*	MK-8	290 µg/g wet cell weight (WCW)	Overexpression of *ispA, dxr, idi* and *menA, menB, menD*; disruption of the competing ubiquinone-8 (Q-8) pathway; anaerobic cultivation [69]
	MK-7	13.6 µM (22-fold improvement)	Introduction of the heterologous MVA pathway; overexpression of HepPPS; blocking the competing Q-7/Q-8 synthesis; aerobic fermentation in 5-L bioreactor [26]
*Bacillus subtilis*	MK-7	69.5 mg/L	Overexpression of *menA, dxs, dxr, yacN*; deletion of *dhbB* to reduce consumption of competing precursor isochorismate [70]
	MK-7	281.4 mg/L (12.0 mg/g DCW)	Overexpression of *glpK, glpD, aroGfbr, pyrGfbr, hepS, vgb*; deletion of *mgsA* and *araM*, adding 10 g/L sucrose into the GSY medium (30 mL/L glycerol, 60 *g*/L soy peptone, 5 g/L yeast extract, 3 g/L K_2_HPO_4_, 0.5 g/L MgSO_4_·7H_2_O, pH 7.3) [32]
	MK-7	50 mg/L	Combined overexpression of the rate-limiting enzymes Dxs and Dxr in the MEP pathway [56]
	MK-7	360 mg/L (shake flask), 200 mg/L (15-L fermenter)	Construct a bifunctional Phr60-Rap60-Spo0A quorum-sensing molecular switch to dynamically regulate fermentation and balance cell growth and product biosynthesis [17]
	MK-7	415 mg/L (shake flask), 242 mg/L (50-L fermenter)	Combinatorial overexpression of five genes in the MEP pathway combined with fermentation condition optimization [15]
	MK-7	Biosynthesis enhanced; specific values not reported	Static culture to form biofilms; modulate membrane composition and electron transport; upregulate oxalate decarboxylase OxdC to produce formate as an electron donor for promoting MK-7 synthesis [18]
	MK-7	226 mg/L	Medium optimization via response surface methodology; elevate agitation speed and aeration; use vegetable oil as an antifoam agent for in situ extractive recovery of MK-7 (80% recovery efficiency) [71,72]

#### 3.3.1 Optimization of culture conditions

Optimization of culture conditions serves as a fundamental approach to synchronously regulate MK profile and production. As reported by Liu et al. [68], MK biosynthesis in *L. lactis* subsp. *cremoris* MG1363 is significantly modulated by the culture conditions, which concurrently change the total yield and MK type. Regarding temperature, MG1363 exhibited the highest total VK2 titer and yield per cell at 30 °C and 33.5 °C, approximately twice that at 37 °C. As the temperature increased from 20 °C to 37 °C, the relative abundance of MK-9 gradually decreased from 80% to 50%, while the proportion of MK-5 to MK-8 increased accordingly [68]. Carbon sources also affect the MK yield and distribution; the relative abundance of MK-9 was approximately 60% when mannitol or galactose was used as the carbon source and approximately 65% when fructose, glucose, trehalose and maltose were used. Notably, the total VK2 titer with mannitol reached 2.4 times that with glucose [68]. It has been suggested that carbon sources might also impact the precursor supply of chorismate, thus changing the production of MK via different carbon metabolic pathways [73]. In contrast, the aeration and energy metabolism modes exert more pronounced regulatory effects; aeration markedly enriches the production of long-chain MKs and greatly boosts total VK2 production, whereas static cultivation favors the accumulation of relatively shorter-chain MKs. Aerobic respiration with heme supplementation further enhanced long-chain MK biosynthesis, significantly increasing the proportions of MK-8 and MK-9, whereas static fermentation resulted in a lower MK-9 ratio and higher levels of medium- and short-chain homologs. Both aerobic fermentation and aerobic respiration significantly increased the VK2 titer and yield per cell compared to static culture. A low oxygen partial pressure in the late growth phase triggers rapid MK accumulation accompanied by a rebound in the short-chain MK proportion, enabling synergistic optimization of the yield and profile by adjusting the culture cycle [68]. As MK biosynthesis is an energy-consuming process, the increase in MK production during aerobic fermentation or aerobic respiration is closely correlated with the greater energy generated under these two conditions.

#### 3.3.2 Strain development

Strain development is a pivotal strategy for simultaneously enhancing MK production and directionally reshaping its profile, either naturally or artificially. Genetic engineering is a feasible strategy for improving MK production in *L. lactis*. Overexpression of *menF* and *menA* promotes MK-3 production, whereas overexpression of *mvk* and *llmg_0196* enhances MK-9 formation [23]. According to the biosynthetic pathway of MK, the overexpression of *menF* could contribute to a greater capacity to produce DHNA, whereas more MenA would increase the prenylation of DHNA through the attachment of isoprenoid side chains. Since long side chains could not be produced in a timely manner, more short-chain MKs (such as MK-3) can be obtained by the overexpression of *menF* and *menA*. Overexpression of *mvk* and *llmg_0196* increases the supply of long side chains, leading to an increase in MK-9 production. Li et al. [25] precisely controlled the biosynthesis of DHNA, the head structure of MKs, by tuning the expression ratio of *menF* to *menD* and optimizing the substrate supply via synthetic biology, maintaining stable production and avoiding cellular toxicity. The knockout of *menF* completely blocked the MK biosynthetic pathway, whereas the heterologous complementation of *menA, menB, menG*, and other genes from *Lactobacillus plantarum* and *Lactobacillus buchneri* can restore aerobic respiration and MK biosynthesis [44,45]. Considering that the practical application of genetically modified organisms in food processing is still limited, laboratory evolution has provided another feasible method for breeding mutant strains with enhanced MK yields. Liu et al. [24] obtained an evolved strain through 100 rounds of subculture and screening under aerobic conditions, which showed a 50–110% increase in long-chain MK production relative to the parental strain, with no obvious shift in the MK profile. Genome sequencing of the evolved strains revealed mutations in *ldh* and *gapB*; however, the underlying mechanisms remain unclear [24]. Moreover, natural variations in MK profile and titer exist among different *L. lactis* strains. For example, Morishita et al. [33] reported *L. lactis* strain YIT 2011 as the main MK-8 and MK-9 producer, which provided beneficial quantities for dietary supplements (i.e., 71 and 113 nmol/L MK-8 and MK-9 in fermented milk, respectively), providing native strain resources for the synergistic production of different MKs.

#### 3.3.3 Remaining key bottlenecks for MK production in *L. lactis*

Even though culture condition optimization and strain development have increased the MK yield in *L. lactis*, several inherent bottlenecks still hinder further yield improvement. The precursor supply can be considered a major limitation. DHNA biosynthesis is initiated by phosphoenolpyruvate and erythrose 4-phosphate, proceeds through the shikimate pathway to yield chorismate, and undergoes seven sequential enzymatic reactions catalyzed by MenF, MenD, MenX, MenC, MenE, MenB, and MenI to produce DHNA. DHNA biosynthesis in *L. lactis* is regulated by the interplay between reversible flux, allosteric feedback inhibition, substrate limitation, and spatial gene arrangement [25]. Static single-gene overexpression only achieves marginal enhancement of DHNA flux and fails to dynamically satisfy precursor demands [25,[31], [70], [74]]. Moreover, uncontrolled DHNA accumulation may generate metabolic stress by perturbing the quinone-mediated redox balance, thereby limiting cellular tolerance toward enhanced VK2 biosynthesis [25,75,76]. Therefore, the existing regulatory strategies in *L. lactis* cannot balance precursor provision and cytotoxicity, becoming the primary constraint on MK biosynthesis [25,70,74]. In contrast, isopentenyl diphosphate serves as a building block for the synthesis of undecaprenyl pyrophosphate, which is an essential substrate for the synthesis of peptidoglycan. As a result, excessive IPP shunted to the side chain of MK affects the normal growth of cells [56,[77], [78], [79], [80]].

MK biosynthesis faces superimposed constraints on saturated membrane loading capacity and intracellular product toxicity. As a highly lipophilic lipoquinone, MK preferentially partitions into the bacterial phospholipid bilayers. However, membrane incorporation capacity is inherently limited, and excessive MK accumulation may disrupt membrane organization and cellular homeostasis [25,[81], [82], [83]]. Unlike many water-soluble metabolites, MK is a hydrophobic, membrane-associated quinone that primarily accumulates within bacterial membranes. Although *L. lactis* possesses a well-characterized genome, no dedicated MK export system has been experimentally identified, making intracellular membrane accumulation the predominant fate of synthesized MK [23,36]. Accumulation of the MK biosynthetic intermediate DHNA mediates feedback regulation of the MK pathway by inhibiting the upstream enzyme MenD [25,76].

The intracellular shortage of NADPH also restricts the efficiency of MK synthesis. Multiple reductive steps in the MK biosynthetic cascade rely heavily on NADPH as a hydride donor. Nevertheless, glycolytic metabolism dominates in *L. lactis*, predominantly generating NADH rather than NADPH, with an extremely low flux through the pentose phosphate pathway. Endogenous NADPH regeneration rates cannot sustain the efficient synthesis of long-chain MK homologs (MK-8/MK-9). Current studies only confirm the elevation of intracellular NADPH pools via the overexpression of glucose-6-phosphate dehydrogenase, while stable dynamic regulatory circuits tailored to replenish the reducing power for MK production remain undeveloped. This leaves a persistent deficit of reducing equivalents for long-chain MK biosynthesis [[34], [84], [85], [86]].

## 4 Strategies for high-level MK production in known cell factories of VK2

*E. coli* and *B. subtilis* are well-known workhorses in biotechnology that produce various metabolites, including VK2. This section summarizes the available strategies for enhancing VK2 production in the two strains (Table 2), providing a reference for overcoming the bottleneck in VK2 production in *L. lactis*.

### 4.1 **High-level production of MK-7 and MK-8 in***E. coli*

As a facultative anaerobic microorganism, *E. coli* is widely employed as an industrial chassis strain because of its rapid growth and ease of genetic manipulation. Strategies for high-level MK production in *E. coli* mainly focus on enhancing precursor synthesis, blocking competing pathways, and implementing heterologous biosynthetic engineering, which provides valuable references for the development of high-yield strategies in *L. lactis*. The quinone profile of *E. coli* exhibits culture condition dependence: ubiquinone-8 (Q-8) is predominantly synthesized under aerobic conditions, whereas MK-8 is the primary product synthesized under anaerobic conditions [69,87]. In 2011, Kong and Lee [69] improved MK-8 production by regulating precursor synthesis and blocking competing pathways. Specifically, the overexpression of IspA (involved in isoprenoid chain elongation), DXR (a rate-limiting enzyme for IPP biosynthesis), and IDI (responsible for the interconversion between IPP and DMAPP) doubled MK-8 production. Moreover, the overexpression of naphthoquinone ring biosynthetic genes, such as *menA* and *menB,* also significantly enhanced the MK-8 yield. Mutation of *ubiC* in the Q-8 biosynthetic pathway increased MK-8 production by 30%, whereas the overexpression of *menA* and *menD* boosted production 5-fold. Finally, a titer of 290 µg/g WCW (wet cell weight) of MK-8 was achieved under anaerobic culture conditions [69]. As *E. coli* cannot naturally synthesize MK-7 owing to the lack of heptaprenyl diphosphate synthase (HepPPS), Gao et al. [26] achieved a breakthrough by introducing the heterologous MVA pathway to supply isoprenoid precursors and overexpressing *B. subtilis*-derived HepPPS, realizing the *de novo* biosynthesis of MK-7 in *E. coli* for the first time. Tuning HepPPS expression increased MK-7 production by 2.3 µM, 22-fold higher than that of the original strain. Deletion of *ubiC, ubiA*, and *ispB* blocked the competing pathways for Q-7 and Q-8 biosynthesis. Ultimately, an MK-7 titer of 13.6 µM (8.8 mg/L) was attained in a 5-L fermenter under aerobic conditions with glucose as the carbon source [26].

### 4.2 **High-level production of MK-7 in***Bacillus***species**

*Bacillus* strains (especially *B. subtilis* and *B. subtilis natto*) are food-grade chassis strains that can undergo convenient genetic manipulation, making them excellent hosts for MK-7 production. High-yield strategies for these strains include modular engineering, metabolic regulation, fermentation optimization, and quorum sensing regulation. As the most favorable host for MK-7 production, *B. subtilis* produces MK-7, which accounts for >90% of its total VK2 yield [30]. Using *B. subtilis* 168 as an example, Yang et al. [70] divided the MK-7 biosynthetic pathway into four modules: the MK-7, shikimate, methylerythritol phosphate, and glycerol metabolic pathways. In another modular engineering study, the MK-7 biosynthetic pathway in *B. subtilis* was divided into five modules. Combined strategies, including the overexpression of pathway genes and feedback-resistant enzymes, deletion of the competing genes *mgsA* and *araM*, and heterologous expression of VHb, were implemented. The final strain BSMK_11 attained an MK-7 titer of 281.4 ± 5.0 mg/L (12.0 mg/g dry cell weight [DCW]) in a 5 L fermenter [32]. Overexpression of MenA, which catalyzes the conjugation of DHNA with the heptaprenyl side chain, yielded 6.6 ± 0.1 mg/L of MK-7 after 120 h of fermentation. Overexpression of 2-C-methyl-d-erythritol 4-phosphate (MEP) pathway genes, including *dxs, dxr*, and *yacN*, also significantly improved MK-7 production. The gene *dhbB* encodes a bifunctional isochorismate lyase/aryl carrier protein that converts isochorismate to (2S,3S)-2,3-dihydro-2,3-dihydroxybenzoate (DHDHB) during bacillibactin biosynthesis and competes for isochorismate, a common precursor with MK-7. The deletion of *dhbB* relieved this precursor competition and reduced intermediate consumption. Consequently, the final engineered strain produced 69.5 mg/L MK-7 in a 2-L baffled flask after 144 h of fermentation [70]. Ma et al. [56] constructed optimal strains by overexpressing different combinations of rate-limiting enzymes, including Dxs and Dxr, which are key rate-limiting enzymes in the MEP pathway that supply isoprenoid precursors for the biosynthesis of the MK-7 side chain. This strategy achieved an 11-fold increase in MK-7 production (up to 50 mg/L) compared to the original strain, confirming that MEP pathway engineering effectively enhanced MK-7 biosynthesis. In addition, Cui et al. [17] constructed a Phr60-Rap60-Spo0A quorum sensing (QS) system to balance cell growth and product synthesis, resulting in MK-7 titers of 360 mg/L in shake flasks and 200 mg/L in a 15-L bioreactor. Chen et al. [15] overexpressed five genes in the MEP pathway, and the engineered strain BS20DFHG produced 415 mg/L MK-7 in shake flasks. After fermentation optimization, the yield reached 242 mg/L in a 50-L bioreactor.

Biofilm formation in static culture promotes MK-7 synthesis by regulating membrane composition and electron transport. In this process, OxdC is upregulated, which generates more formate as an electron donor to enhance MK-7 synthesis [18]. During the biosynthesis of MK-7 in *B. subtilis*, the specific productivity and effective product accumulation in stirred bioreactors were significantly lower than those in shake flasks. This is attributed to high hydrodynamic shear stress, microenvironmental heterogeneity, metabolic flux redistribution, loss of lipophilic products, and inadequate scale-up strategies, resulting in a typical scale-up attenuation phenomenon, in which bioreactor titers are inferior to the shake flask results. High-yield MK-7 production by *B. subtilis natto* can also be achieved through fermentation optimization. After medium optimization, the yield reached 62.32 ± 0.34 mg/L, which increased to 226 mg/L under high-speed stirring and aeration. Using vegetable oil as an antifoaming agent, MK-7 was recovered in situ at a recovery rate of 80%, establishing a one-step process for producing MK-7-enriched vegetable oil that can be directly used in food production, further enhancing the high-yield application strategies of *Bacillus* spp [71,72].

### 4.3 Perspectives for the improvement of VK2 production in *L. lactis*

Although engineering strategies in *E. coli* and *B. subtilis* have significantly enhanced VK2 production, these specific modification schemes cannot be directly transplanted into *L. lactis* because of interspecies differences in fundamental physiological and genetic traits, constituting the core intrinsic cause for the poor universality of cross-chassis engineering tactics. Principally, *L. lactis* has undergone reductive genome evolution in long-term dairy niches, losing numerous genes related to carbon flux branching, which renders its highly streamlined metabolic network with low metabolic redundancy and a far weaker capacity to buffer metabolic perturbations relative to *B. subtilis* and *E. coli*. The heterologous introduction of multigene synthetic modules, such as the MEP pathway, which is absent in *L. lactis*, might trigger carbon metabolic imbalance and growth arrest. Therefore, the established engineering strategies for *E. coli* and *B. subtilis* provide valuable references for improving MK production by *L. lactis*.

First, genes responsible for competing metabolic branches, including lactic acid and branched-chain amino acids, can be accurately blocked to channel the intracellular carbon flux toward MK synthesis and boost precursor pools at the carbon-partitioning level. At the same time, blocking the lactic acid pathway results in the accumulation of NADH produced during glycolysis; thus, NADH might be transformed into NADPH under the action of the transhydrogenase PntAB [88]. Second, rate-limiting genes such as *menD* and *menF* can be site-specifically overexpressed by tunable promoters to alleviate the flux imbalance between DHNA precursor and isoprenoid side chain synthesis. Simultaneously, scarless point mutations can be introduced to modify the functional domains of biosynthetic enzymes, effectively enhancing the catalytic activity of key enzymes and overall pathway flux to relieve rate-limiting barriers. Third, modular pathway engineering can split the upstream shikimate pathway responsible for DHNA formation and the MVA pathway, providing isoprenoid side chains into separate expression modules to simultaneously increase the carbon flux through both precursor branches and break the MK production bottleneck. Fourth, dynamic control can be applied to balance cell growth and MK synthesis from the perspective of IPP demand, as demonstrated in *B. subtilis* using QS systems [17]. Moreover, a successful attempt to engineer cell membranes for the accumulation of hydrophobic terpene products could provide a new direction for engineering and improving microbial MK producers [89].

Genetic manipulation tools are indispensable for the smooth implementation of possible metabolic pathway modifications in *L. lactis*. Over the past few decades, a variety of genetic engineering strategies, including plasmid-based expression systems, homologous recombination approaches, and advanced genome-editing technologies, have been developed for lactic acid bacteria, greatly expanding their applications in synthetic biology and metabolic engineering. Existing reviews have elaborated classical genetic modification tools for *L. lactis* [90]. In *L. lactis*, conventional genetic modification approaches have primarily relied on homologous recombination-based strategies, which have provided a foundation for functional genomics and metabolic engineering; however, they generally require extensive screening procedures and are unsuitable for complex genome engineering [90]. Recent advances in genome-editing technologies, particularly CRISPR-Cas9-based systems, have significantly expanded the genetic toolbox available for *L. lactis* engineering [91,92]. By introducing sequence-specific DNA cleavage and facilitating donor DNA-mediated homologous recombination, CRISPR-Cas9 enables rapid, programmable, and markerless genome editing, including targeted gene deletions, insertions, and point mutations [91,93]. Optimized CRISPR-Cas9 platforms have been successfully established in representative *L. lactis* strains, including MG1363 and NZ9000, enabling efficient genome modification with a simplified workflow [91,92]. Recently, a combination of CRISPR/Cas9-assisted single-stranded DNA recombineering with the Cre/loxP system achieved large DNA integration without residual selection markers in *L. lactis* [94]. Moreover, given the restrictions on genetically engineered strains in the food industry, natural horizontal gene transfer modes have regained much attention for obtaining non-genetically engineered strains that might be used in practical applications. For example, two conjugation systems have been identified in the lactococcal plasmids pNP40 and pUC11B [95,96]. After an optimized conjugation protocol, the transfer frequency was approximately 4000-fold higher than that reported previously [95]. Another notable natural horizontal gene transfer mode is the natural transformation of *L. lactis*. In some *L. lactis* strains, the master competence regulator ComX and the complete set of proteins involved in natural transformation have been identified [97,98]. Through the constitutive overproduction of ComX, natural DNA transformation can be functional in *L. lactis* [98]. Collectively, both advanced genome engineering tools and potential horizontal gene transfer modes would provide a robust foundation for the precise pathway rewiring and metabolic optimization of *L. lactis*.

## 5 Summary

Vitamin K2 (MK-n), a member of the lipid-soluble vitamin family that is key to bone homeostasis and vascular health with long-chain MKs (n = 5–13), has better bioavailability than short-chain MKs. Developing vitamin K2-enriched fermented foods has great theoretical and practical significance, of which fermented dairy products can serve as excellent carriers of VK2. *L. lactis* is an emerging food-safe chassis for producing long-chain MKs; however, compared to *B. subtilis, L. lactis* exhibits a significantly lower VK2 production yield. Recently, through the optimization of culture conditions, adaptive laboratory evolution, and simple metabolic engineering modifications, VK2 production has increased to some extent. In the future, blocking competing pathways, identifying rate-limiting steps, decoupling of cell growth, and improvement of MK synthesis will contribute to enhancing VK2 production in *L. lactis*.

## Data availability

Data will be made available on request.

## CRediT authorship contribution statement

**Yu Lou:** Writing – original draft, Visualization. **Jingjing Shuang:** Writing – original draft. **Jian Kong:** Supervision, Resources. **Tingting Guo:** Writing – review & editing, Supervision, Project administration, Conceptualization.

## Declaration of competing interest

The authors declare that they have no known competing financial interests or personal relationships that could have appeared to influence the work reported in this paper.
